# Enhancement of polymer thermoresponsiveness and drug delivery across biological barriers by addition of small molecules

**DOI:** 10.1016/j.heliyon.2023.e16923

**Published:** 2023-06-07

**Authors:** Zipei Zhang, Xiyu Li, Changwoo Do, Daniel S. Kohane

**Affiliations:** a Laboratory for Biomaterials and Drug Delivery, Department of Anesthesiology, Division of Critical Care Medicine, Boston Children's Hospital, Harvard Medical School, Boston, MA, 02115, USA; b Neutron Scattering Division, Oak Ridge National Laboratory, Oak Ridge, TN, 37831, USA

**Keywords:** Thermoresponsive polymers, Chemical permeation enhancer, Gelation, Tympanic membrane, Otitis media

## Abstract

Thermoresponsive polymers that undergo sol–gel transitions in the physiological temperature range have been widely used in biomedical applications. However, some commercially and clinically available thermoresponsive materials, particularly poloxamer 407 (P407), have the significant drawback of insufficient gel strength, which limit their performance. Furthermore, co-delivery with some small molecules, including chemical permeation enhancers (CPEs) can further impair the physical properties of P407. Here, we have developed a thermoresponsive platform by combination of CPEs with the poloxamer P188 to enable gelation at physiological temperatures and enhance gel strength. P188 gels at 60 °C, which is far above the physiological range. In combination with limonene (LIM) and sodium dodecyl sulfate (SDS), P188 gels at ∼25 °C, a temperature that in useful for biomedical applications. Gelation behavior was studied by small angle neutron scattering (SANS) experiments, which identified micelle-to-cubic mesophase transitions with increasing temperature. Analysis of the SANS intensities revealed that P188 micelles became larger as LIM or SDS molecules were incorporated, making it easier to form a micellar gel structure. P188-3CPE (i.e., 2% LIM, 1% SDS and 0.5% bupivacaine (BUP)) had low viscosity at room temperature, facilitating administration, but rapidly gelled at body temperature. P188-3CPE enabled the flux of the antibiotic ciprofloxacin across the TM and completely eradicated otitis media from nontypable Haemophilus influenzae (*NTHi*) in chinchillas after a single administration.

## 1 Introduction

Materials with reverse thermal gelation (here referred to as thermoresponsive gels) are important in the context of many types of drug delivery applications, topical and injected [1,2]. Poloxamer 407 (P407), a commonly used example of such a thermoresponsive material [[3], [4], [5], [6]], is an ABA block copolymers where A = poly(ethylene oxide) and B = poly(propylene oxide). However, P407 has low gel strength, with weak mucoadhesiveness and rapid gel dissolution, which has limited its performance in drug delivery [7]. The gel strength of P407 has been improved by conjugation with other polymers or by introducing inter-micelle linkers to the terminal hydroxyl groups [[8], [9], [10], [11], [12]]. The challenge is to improve the physical properties of the hydrogel without expensive and time-consuming preparation procedures [13], and without compromising the desirable mechanical and drug-delivery properties.

Applicability through a small gauge applicator is desirable in many clinical contexts and requires low viscosity. Once applied, usually into an environment at body temperature (37 °C), it is important for the material to gel rapidly and with adequate mechanical strength, to avoid removal of the drug delivery system from the site of placement, and to enhance control of drug release [14,15]. Tissue penetration of drugs from such systems can be enhanced by chemical permeation enhancers (CPEs), compensating for the tendency of more viscous systems to slow drug release. Unfortunately, the gelation properties of P407 are impaired by addition of small molecules such as CPEs, a problem that has been addressed by modification with hydrophobic phosphoester groups [16].

Here we have built on the fact that CPEs can alter the rheological properties of poloxamers and their derivatives [17,18] to develop a thermoresponsive poloxamer-based gel with strong mechanical properties in the presence of CPEs, while maintaining low viscosity at room temperature and rapid gelation, and providing therapeutically effective drug flux. We elucidate the effects of the CPEs on the ultrastructure of the gel to explain their effects on the mechanical properties.

To demonstrate the effectiveness of this approach, we selected an animal model of a clinical context where the thermosensitive properties of the formulation are important, as is the enhancement of drug flux with CPEs: the non-invasive delivery of antibiotics across the tympanic membrane (TM) into the middle ear in otitis media. We have previously described a therapeutic platform comprising a thermoresponsive gel, chemical permeation enhancers, and the antibiotic ciprofloxacin. A single dose administered on the TM could eradicate otitis media (OM) in a chinchilla model [19]. There, the obvious challenge is in getting drugs across a barrier – the tympanic membrane (TM) - that has very low permeability to most molecules. However, additional design constraints are created by the nature of the target patient population - most of whom are less than two years old and are therefore unlikely to stay still for extended periods – and by the need to provide therapy for approximately one week. Consequently, the drug delivery system has to have low viscosity at room temperature so that it can be applied easily and then flow down the external auditory canal to the TM, it has to gel rapidly upon contact with the warm tympanic membrane and have mechanical properties strong enough that it remains at the TM to provide treatment over several days. Moreover, the gel cannot be so viscous that it slows efflux of the drug to the point that flux across the TM is inadequate.

## 2 Results

### 2.1 Rheological properties

**Fig. 1:**
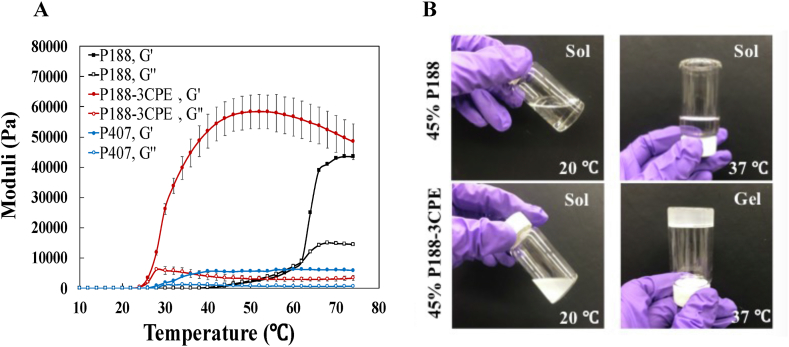
(A) Mechanical properties of 45% P188, 45% P188-3CPE, and 18% P407, as a function of temperature (G′ and G″ represent the storage and loss moduli, respectively). 3CPE: 2% LIM, 1% SDS and 0.5% BUP. Comparison to 18% P407 showed that CPEs lowered the gelation temperature of P188 to below that of P407. Data are means ± SD (*n* = 4). (B) Images of 45% P188 and 45% P188-3CPE at room or body temperature.

**Fig. 2:**
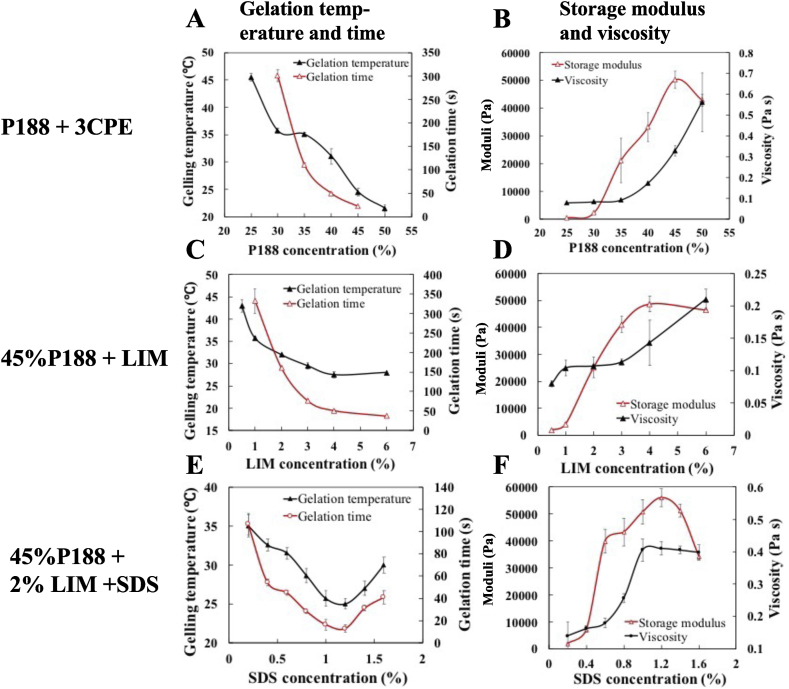
Physical properties of the formulations. A, B. Various concentrations of P188 with 3CPE (2% LIM, 1% SDS and 0.5% BUP). C, D. 45% P188 with various concentrations of LIM. E, F. 45% P188 with various concentrations of SDS in presence of 2% LIM. The effect of SDS alone is not shown as it prevented gelation (Figure S3A). Physical properties of the formulations included: gelling temperature, gelation time, viscosity at room temperature (at 100 s−1 shear rate), storage modulus (G′) at 37 ℃ and viscosity at room temperature. Data are means ± SD (*n* = 4).

To identify poloxamers whose mechanical properties might be enhanced by CPEs, a screen (Figure S1 and S2) was conducted of the rheological properties of a range of common commercially available poloxamers (i.e., P101, P124, P181, P182, P231, and P338), with and without the addition of a previously described synergistic combination [20] (termed 3CPE) of the CPEs 2% limonene (LIM), 1% sodium dodecyl sulfate (SDS) and 0.5% bupivacaine (BUP). P407 was not studied as it had already been established that CPEs impair its physical properties [21]. None of the poloxamers showed thermoresponsive gelation properties with or without CPEs, except for P188, which had a sol-gel transition temperature >60 °C at a range of concentrations (Figure S1 and S2). Surprisingly, the addition of 3CPE to 45% P188 sharply decreased the gelation temperature to ∼ 25 °C (Fig. 1A and B), rendering it usefully thermoresponsive in the physiologic temperature range. 45% P188 did not gel below 25 °C, i.e., at room temperature. 45% P188 was used in subsequent experiments with CPEs because of these gelation properties, and because the highest G’ was obtained at that concentration (Fig. 2B).

At room temperature (∼20 °C), 45% P188-3CPE was a liquid with low viscosity (∼ 0.3 Pa s) (Fig. 2B), which could be easily drawn up and extruded through a 20-gauge catheter. After submersion in a 37 °C water bath 45% P188-3CPE gelled in ∼13 s.

We studied the physical properties of 45% P188 in the presence or absence of different CPE combinations as a function of the concentrations of the individual components. In the presence of 3CPE (i.e., P188-3CPE), increasing the P188 concentration (Fig. 2A and **B**) decreased the gelation time and gelation temperature, but increased the viscosity at room temperature and storage modulus (G′) at body temperature. In the absence of 3CPE, increasing the LIM concentration with 45% P188 reduced the gelation time and temperature, and increased room temperature viscosity and G’ (Fig. 2C and **D**). In contrast, the addition of 1% SDS (in the absence of other CPEs) completely suppressed the reverse thermal gelation behavior of P188 (Figure S3A). However, the addition of SDS to P188-2% LIM enhanced gelation (i.e., lowered gelation time and temperature, increased G’) at < 1.2% SDS, but inhibited gelation at higher concentrations (Fig. 2E and F).

### 2.2 Structural properties of P188-CPE mixtures

We used small angle neutron scattering (SANS) to explore the effect of CPEs on formation and structural changes of P188 micelle to understand how CPEs lowered the gelation temperature of P188 from >60 °C to ∼25 °C. The gelation of poloxamers has been generally attributed to the formation and subsequent packing of polymer micelles.

**Fig. 3:**
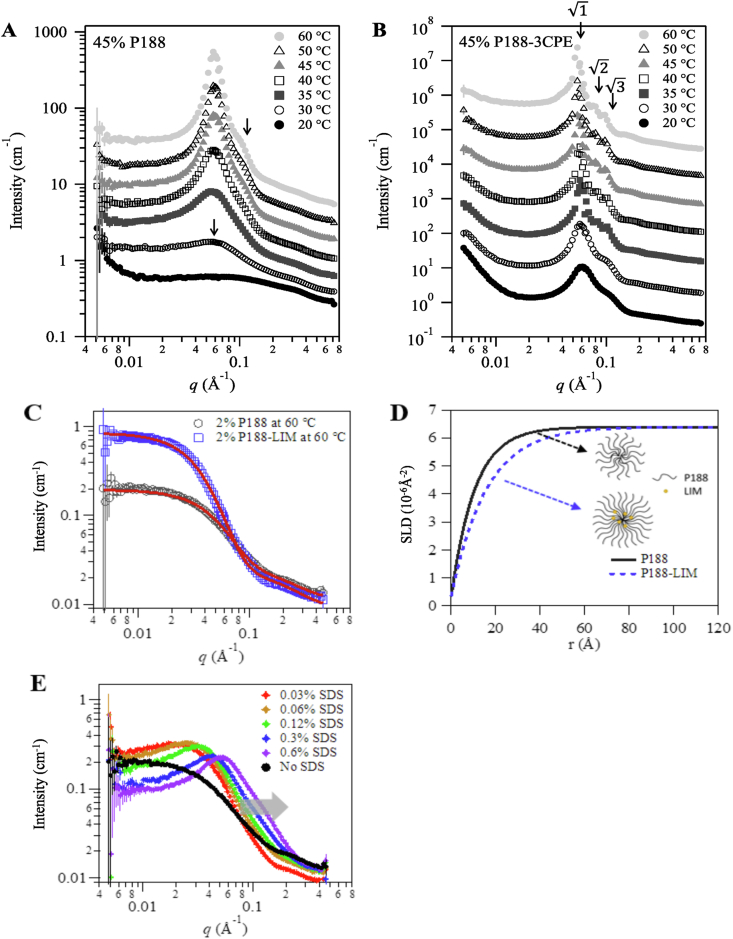
SANS scattering intensities from (A) 45% P188. (B) 45% P188-3CPE, arrows correspond to the peak positions at 0.058, 0.084 and 0.102 Å−1 (the “ : : ” pattern). Panels A and B are at 20–60 ℃. (C) 2% P188 with/without 0.12% LIM (Solid red lines show the fits according to the spherical micelle model fits). (D) SLD profiles obtained from the model fitting of 2% P188 with/without LIM at 60 ℃. (E) 2% P188 with various SDS concentrations at 60 ℃. 3CPE: 2% LIM, 1% SDS and 0.5% BUP. Arrow indicates the shift of scattering profiles to higher *q* values.

Samples of 45% P188 were measured by SANS at various temperatures (Fig. 3A). As temperature increased, a broad peak around *q* = 0.06 A^−1^ appeared at around 30 °C, then became sharper and had greater intensity above 40 °C. Transitioning to a sharper and stronger scattering peak typically indicates the appearance of structural ordering at a length scale of 2π/*q*, where *q* is the position of the peak. In this case, the characteristic length scale for such structural order is about 105 Å. Above 40 °C, a shoulder peak was observed at around *q* ≈ 0.1 Å^−1^, which is approximately √3 times the *q* value of the first peak (∼ $0.06\times \sqrt{3}$ = 0.104 Å^−1^) indicating development of a liquid crystalline phase [22]. The observed changes of the scattering intensity as a function of temperature are consistent with the known phase behavior of P188 in water, which shows a monomer-micelle-cubic liquid crystalline phase transition with increasing temperature [[23], [24], [25]]. The slight increase of the moduli at 40 °C (Fig. 1A) corresponds to the onset of gel formation observed from the scattering intensity, and the formation of a cubic liquid crystalline phase accounts for the increasing moduli at higher temperatures. With the addition of 3CPE to 45% P188, formation of the liquid crystalline phase was visible at 20 °C (Fig. 3B), and the main scattering peak appeared around *q* ≈ 0.06Å^−1^, which was similar to the peak position observed for pure P188. The unchanged *q* position of the scattering peak for 3CPE-P188 compared to P188 suggests that 3CPE lowers the temperature at which the onset of structural or phase transition of P188 solution happens, but does not change the characteristics of the microstructure observed from pure P188 solution. Higher order peaks became clearly visible above 35 °C, indicating formation of a highly ordered liquid crystalline phase (Fig. 3B). The relative peak positions observed from the scattering intensities above 35 °C had ratios of $\sqrt{1}$: $\sqrt{2}$: $\sqrt{3}$ for peaks at *q* ≈ 0.0578, 0.08, and 0.102 Å^−1^, suggesting supramicellar cubic structures [19,26]. While the practically identical scattering peak positions observed in P188+3CPE suggests that the structure is similar to the one observed from pure P188 solution, the much narrower peaks for P188+3CPE compared to the corresponding peaks for pure P188 indicates that the degree of structural ordering in the cubic gel phase had been enhanced significantly by addition of 3CPE [19].

In order to understand the role of 3CPE in enhancing the formation of micelles and cubic superstructure of micelles, we investigated the structure of low concentrations of P188 (i.e., 2% or 5%) solution with and without CPEs, i.e., (SDS or LIM) [[23], [24], [25]]. BUP was not investigated since it had a minor effect on the rheological and gelation properties of P188 (Figure S3B). The structural changes observed in micelle formation may shed light on the formation of gels at higher concentration of P188 with 3CPE. In Fig. 3C, the scattering intensities of 2% P188 solution were compared to those from P188-LIM at 60 °C. The addition of LIM enhanced the scattering intensity which may be the result of formation of bigger micelles or increased number of micelles. Both scattering intensities were fitted with spherical micelle models with smoothly decaying scattering light density profiles (SLD; see description in **Supplementary Information**), assuming the SLD of the core and the solvent do not change (Fig. 3C). The SLD value is a measure of the scattering power of a material and typically increases with the physical density and intrinsic scattering power of the scattering entities, i.e., the number of molecular species per unit volume. Micelles have a distribution of SLDs, termed the SLD profile (Fig. 3D), which reflects the distribution of atoms as a function of a distance from the center of the micelle, i.e., micelle size. Here, it was assumed that solvent molecules (in this case water) do not exist at the hydrophobic core of the micelle (SLD of P188 or LIM <0.5 × 10^−6^ Å^−2^, calculated). As the distance from the center of the micelle increases, the SLD value of the micelle becomes that of water (6.34 × 10^−6^ Å^− 2^, calculated), at the end of the P188 chain, and is the size of the micelle or its corona. The SLD values of P188-LIM micelles are lower than those of pure P188 micelles at any given distance from the center of the micelles (Fig. 3D). This suggests that at any given distance from the center of the micelles, there is more polymeric mass in P188-LIM than P188 micelles, i.e., the core and corona of P188-LIM micelles are bigger. This may be ascribed to LIM promoting micellization of P188 by being incorporated within the cores of the micelles that are forming from the hydrophobic block (PPO) of the P188. This view is supported by our previous observation that condensation of a similar type of block copolymer, PE9400, is enhanced at the limonene-water interface [27]. Increased overall size of P188-LIM micelles will encourage formation of a percolated gel phase by increasing the effective volume fraction of polymer micelles [28,29], making it easier for neighboring micelles to be connected, therefore promoting the formation of a liquid crystalline cubic phase as observed in Fig. 3B.

Studies of the association between SDS and PEO-PPO-PEO type block copolymers [30,31] have suggested that low concentration of SDS molecules are incorporated into block copolymer micelles, resulting in charged micelles. As the concentration of SDS increases, the micelle becomes more SDS-rich, resulting in effectively smaller micelles and in the formation of larger number of SDS micelles. The SANS data from P188 mixed with various concentration of SDS at 60 °C (Fig. 3E) also qualitatively show these trends. At 0.03% SDS, broad structure factor peaks around *q* ~ 0.03 A^−1^ appear, indicating formation of charged micelles. As the concentration of SDS increased, the scattering profiles shifted to higher *q* values, indicating micelles becoming smaller and the number of micelles increasing. The reduction in micelle size with increasing SDS concentration was also seen by dynamic light scattering (DLS) with 5% P188 (Figure S4). This structural change observed from SANS may also explain the decreasing moduli and viscosity with increasing SDS concentration above 0.8%. It should be noted that the P188 concentration in the rheology studies were much higher than those used in the SANS studies. What the SANS experiment suggests is that above certain concentrations of SDS, micelles will become smaller as a function of SDS concentration. The ratio of SDS to P188 in the SANS study (0.03% SDS with 2% P188) and the rheological study (∼0.7% SDS with 45% P188) were similar (both ∼0.016). This is close to the concentration of SDS (∼0.9%) at which the viscosity starts to decrease in Fig. 2E. Increasing the SDS/P188 ratio above 0.03/2 = 0.016 decreased the P188-SDS micelle size, making it difficult to form a percolated gel structure. Formation of a gel phase may also have been prevented by the micelles being charged and having Coulomb repulsion [13,32].

### 2.3 *Trans*-tympanic drug flux from the hydrogel

**Fig. 4:**
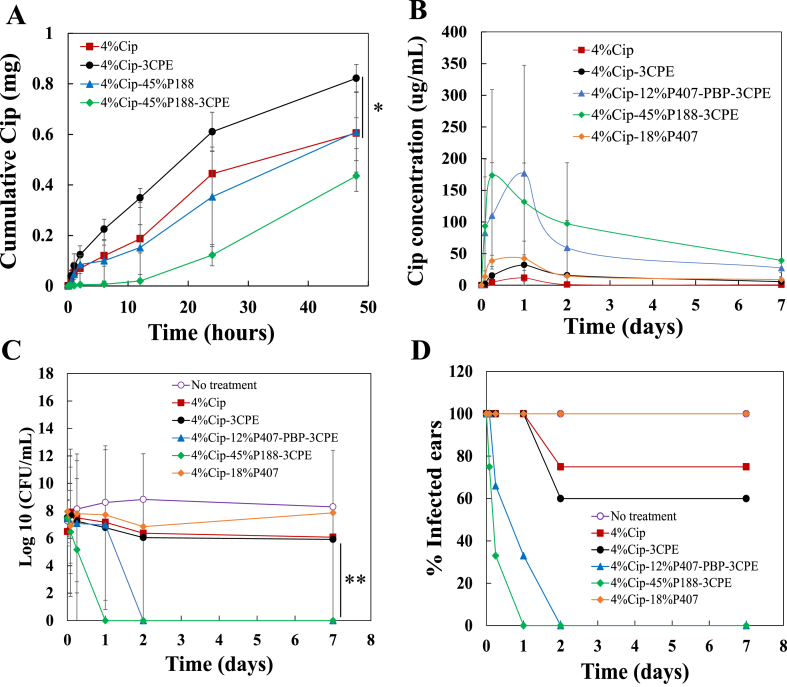
Application of formulations at the tympanic membrane (TM). (A) Cumulative Ex vivo transfers of ciprofloxacin (Cip) across the TM (*n* = 4). (B) Time course of concentration of Cip in middle ear fluid in animals with otitis media (OM) from NTHi treated with different formulations containing 4% Cip (*n* = 5), 4% Cip-3CPE (*n* = 5), 4%Cip-3CPE-P407-PBP (*n* = 5), 4%Cip-3CPE-45% P188 (*n* = 6) and 4% Cip-18%P407 (*n* = 5). (C) Time course of bacterial CFU in middle ear fluid in animals with OM from NTHi treated with different formulation. (D) Percentage of animals with OM (defined as nonzero CFU values in their middle ear fluid aspirates) after administration of different formulations. 3CPE: 2% LIM, 1% SDS and 0.5% BUP. PBP: polybutylphosphoester. Data are means ± SD.

We investigated the effects of P188-3CPE on the flux of ciprofloxacin (Cip) across the TM, ex vivo. In healthy TMs excised from chinchillas (Fig. 4A), 3CPE increased the 48-h flux of Cip from 4% Cip solution from 0.6 mg to 0.82 mg (*P* < 0.05) and the flux from 4% Cip-45% P188 from 0.43 mg to 0.61 mg. The hydrogel itself (4% Cip-45% P188-3CPE or 4% Cip-45% P188) decreased the Cip flux across the TM. The enhancement of flux by 3CPE compensated for the decrease in flux from the hydrogel, so that the flux of drug from 4% Cip-45% P188-3CPE was comparable to that from 4% Cip (Fig. 4A).

### 2.4 Treatment of otitis media with the hydrogel formulation

OM was induced in chinchillas by direct inoculation of non-typable *Hemophilus influenzae* (*NTHi*) into the middle ear, then the infected chinchillas were treated with different test formulations deposited on the TM via the outer ear canal. Middle ear fluid (MEF) was sampled at predetermined intervals for drug content and number of colony-forming units (CFU) of bacteria. In animals with OM, a 4% Cip solution resulted in little *trans*-tympanic flux across the TM (Fig. 4B), which was only slightly improved by the presence of 3CPE, presumably because these two formulations did not remain at the TM. In animals treated with 4% Cip-45% P188-3CPE, the concentration of Cip in MEF rose rapidly, peaked at 173.47 μg/mL at 6 h then declining gradually. Even at one week after placement of the gel, the Cip concentration was 39.125 μg/mL, which is at least 80 times the minimum inhibitory concentration (MIC) for *NTHi* (0.1–0.5 μg/mL) [33,34], and is at least ten times the MIC for another bacterium that commonly causes OM, *S. pneumoniae* (0.5–4 μg/mL). These results indicated that the hydrogel formulations could remain at TM as a reservoir to deliver drug across the TM. The middle ear fluid ciprofloxacin peak concentration from 4% Cip-18%P407 (42.35 μg/mL) was lower (*P > 0.05*) than from 4% Cip-45% P188-3CPE.

In the absence of treatment, bacterial counts in middle ear fluid in animals with OM remained elevated for the full 7 days of observation (Fig. 4C), as was the case in animals treated with 4% Cip and 4% Cip-3CPE. Bacteria were cleared from MEF within 24 h in animals treated with 4%Cip-45% P188-3CPE and no bacteria could be detected even after 7 days of treatment. In contrast, the average number of colonies (CFU/ml) in middle ear fluid remained high (*P* < 0.01) in animals treated with 4% Cip and 4% Cip-3CPE. These results were consistent with the time course of ciprofloxacin levels in the middle ear of animals treated with the various formulations (Fig. 4B). Similarly, resolution of OM (defined as zero colony-forming units (CFUs)) in the MEF was seen at 24 h with 4% Cip-45%P188-3CPE (Fig. 4D).

The performance of P188 was compared to that of a previously reported polybutylphosphoester-modified P407 (P407-PBP) in treating otitis media [16]. 4% Cip-45% P188-3CPE performed as well as or better than 4% Cip-12% P407-PBP-3CPE in the peak level of Cip in middle ear fluid and the rapidity of reaching that peak (Fig. 4B), and clearing the infection (Fig. 4C and D). The 4% Cip-45%P188 (no CPE) was not studied in these experiments since P188 did not gel at body temperature in absence of CPEs and therefore would not remain at the TM. We have previously shown that gelation on the TM is necessary for such systems to have a therapeutic effect [16].

### 2.5 Biocompatibility of hydrogels and systemic drug level

**Fig. 5:**
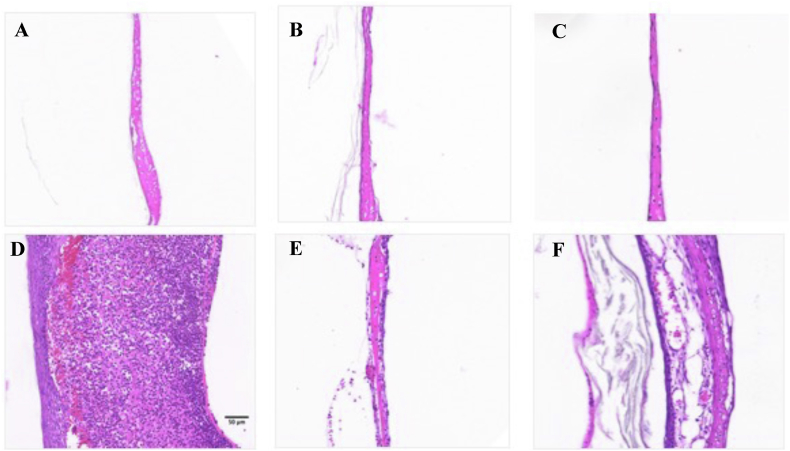
Tissue reaction to different formulations in vivo. H&E-stained sections were obtained from healthy TMs (A) without treatment, (B) treated with 4% Cip-45% P188-3CPE, or (C) treated with 4% Cip (Cip) and TMs after 7 days of otitis media (D) without treatment, (E) treated with 4% Cip-45% P188-3CPE or (F) treated with 4% Cip (Cip).

P188-3CPE remained on the TM for up to 21 days in chinchillas without OM (Figure S5). The histological appearance of TMs (Fig. 5) was assessed on H&E-stained sections after 7 days of treatment with 4% Cip-45% P188-3CPE, in the presence or absence of OM, and compared to TMs in untreated animals with or without OM. In animals without OM, TMs with and without treatment with 4% Cip-45% P188-3CPE appeared similar (i.e., there was no inflammation or tissue injury). In animals with OM, the TM was markedly swollen and inflamed; this returned to normal with treatment with 4% Cip-45% P188-3CPE but not with treatment with Cip alone.

### 2.6 Systemic distribution of ciprofloxacin

One important advantage of transtympanic formulations is that the drug can be directly delivered to the target site, which can avoid the side-effects induced by systemic antibiotic distribution. We investigated the blood levels of Cip at predetermined intervals (Table 1) by serial blood draws from the transverse sinuses of chinchillas with OM after hydrogel treatment. No Cip was detectable in the bloodstream by HPLC at any time point throughout the course of treatment.

**Table 1:** Systemic exposure to ciprofloxacin. Blood samples were analyzed for ciprofloxacin content by HPLC at predetermined intervals after gel application (i.e., 4% Cip-45% P188-3CPE).

Time	Concentration of ciprofloxacin in plasma (μg)
0	Not detected
2 h	Not detected
6 h	Not detected
1 day	Not detected
2 days	Not detected
7 days	Not detected

## 3 Discussion

A key objective of this approach was to enhance the physical properties of the hydrogel, not have them be weakened by addition of CPEs, and have effective drug delivery – without requiring synthetic or other complicated procedures. Here, CPEs altered the thermoresponsive gelation properties of P188 by decreasing its gelation temperature from >60 °C to ∼25 °C, rendering it a useful platform for local drug delivery. The commonly used poloxamer, 18% P407, has a similar gelation temperature (∼25 °C) to 45% P188-3CPE [35]. However, the 45% P188-3CPE showed much greater gel strength (G’ ∼45 k) at 37 °C than a commonly used poloxamer 18% P407 (G’ ∼6 k). This enhanced gel strength could be advantageous in drug delivery applications, prolonging the duration of a depot. As seem here, and noted previously [16], the mechanical properties are important to outcome: the liquid formulations did not cure OM, while the gel did. Moreover, the efficacy of 4% Cip-45% P188-3CPE in treating experimental OM was comparable to that from a synthetic modification of P407, 4%Cip-12% P407-polybutylphosphoester [16].

**Fig. 6:**
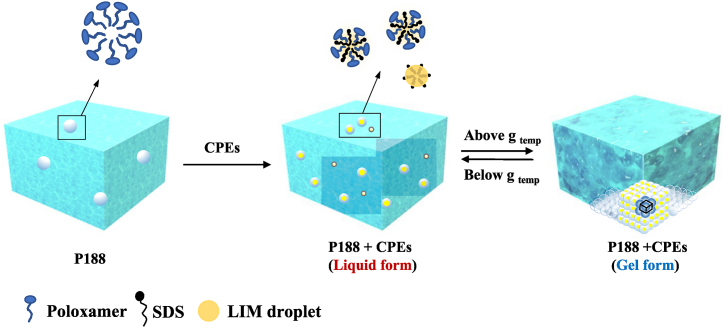
Schematic illustration of the mechanism of adding CPEs promote the gelation of P188.

The CPEs had different effects on gelation properties of P188. Overall, LIM promoted gelation, while SDS suppressed it. In the presence of LIM, however, SDS could also promote the gelation of P188-LIM at low concentrations. We have explored the ultrastructure of P188 by using SANS in presence of different CPEs. Measured SANS intensity profiles showed that the characteristic scattering peak position is unchanged for 3CPE-P188 compared to pure P188 (i.e., 45%) but lowers the temperature at which the onset of structural or phase transition of P188 solution happens. The inclusion of LIM into the hydrophobic core of P188 micelle made the corona of the micelles extend further, which encouraged neighboring micelles to connect. On the other hand, incorporation of SDS with P188 polymers lead to smaller micelles with higher number density within the investigated concentration range. It has been demonstrated that poloxamers can form stiff gels with a liquid crystalline cubic structure once a critical amount of poloxamer micelles (effective volume fraction φ = 0.53) are formed and packed together [13]. Therefore, the effect of SDS should be concentration dependent. At low concentrations of SDS, the addition of SDS to P188-LIM promoted gelation, because the hydrophobic LIM loaded into the micelle core contributed the increase of the micelle size, so that the overall φ increased due to high number density of new formed P188-SDS micelles. This is the reason that CPEs lowered the gelation temperature of P188 as observed in our study (Fig. 6). At higher SDS concentrations, the size of P188-SDS aggregates decreased so that the contribution of the P188 micelles to φ became significantly lower, which could not be compensated by the increased number density of P188-SDS micelles [13]. Hence, the overall φ was decreased and gelation was inhibited at higher SDS concentration as indicated by the rheological studies in Fig. 2.

The rheological properties of P188 remained largely unchanged by the bupivacaine concentration up to 4% (a very high and saturating concentration). This is beneficial in that bupivacaine is a drug in addition to being a CPE. We have previously demonstrated that CPEs enhance BUP flux across the intact TM. The amount of BUP flux across the TM was related to the initial drug loading [36]. Our finding here suggests that even high bupivacaine concentrations would not interfere with gelation.

With 4% Cip-45% P188-3CPE, the peak drug concentration achieved by one dose of hydrogel was more than thousand times the MIC for *NTHi* and more than 300 times the MIC for another more resistant OM-causing otopathogen (i.e., *S. pneumoniae*). After one week of hydrogel placement, the drug concentration in the middle ear was still more than 350 times and 10 times the MICs for *NTHi* and *S. pneumoniae*, respectively. Nevertheless, the antibiotic was undetectable in the bloodstream by HPLC, suggesting no systemic exposure of drug. Systemic exposure is particularly important with OM, due to its high prevalence and recurrence rate, which is believed to be partially responsible for the development of antibiotic-resistant pathogenic bacteria [37].

## 4 Experimental section

**P188 formulation preparation.** A certain amount of P188 was dissolved in deionized water (Milli-Q purification system, Millipore) to prepare poloxamer solution. The SDS and bupivacaine were sequentially added in the poloxamer solution in cold room and stirred at 4 °C for 12 h. Then the limonene was added into the mixture solution and stirred at 4 °C for at least 2 h to obtain the final formulation contain 45% P188, 2% limonene, 1% SDS and 0.5% bupivacaine. The concentration in gels is expressed as the mass/volume percentage (w/v), unless specified otherwise. The CPEs concentrations were selected based on those reported to be effective and minimally toxic in previous transdermal studies [38].For the drug loaded formulation, the powdered P188 was dissolved in ciprofloxacin solution and then the CPEs were sequentially added as described above. Hydrogel formulation in scintillation vials was immersed in a water bath kept at 37 °C, and the time it took for the hydrogel to firmly stick in the bottle without flow away after overturn the vial was recorded as the gelation time. The effects of different composition on the formation and mechanical properties of the hydrogels were investigated by rheological measurements.

**P407-PBP formulation preparation**. The poloxamer 407–polybutylphosphoester (P407-PBP) was synthesized based on our previous methods [16].Typically, 2-Chloro-2-oxo-1,3,2-dioxaphospholane (COP) (5.0 g, 35 mmol) in anhydrous THF was added to a stirring solution of n-butanol or 2-ethyl-1-butanol (2.6 g, 35 mmol) and trimethylamine (TEA, 3.9 g, 39 mmol) in anhydrous THF at 0 °C dropwise. The reaction mixture was stirred in an ice bath for 12 h upon completed addition. The reaction mixture was filtered, and concentrated filtrate was purified by vacuum distillation under reduced vacuum to get the Butoxy-2-oxo-1,3,2-dioxaphospholane (BP). P407 (8.1 g, 0.56 mmol) and BP (1.0 g, 5.6 mmol) in anhydrous dichloromethane (DCM; 0.5 ml) were mixed in a flame dried Schlenk flask and flushed with nitrogen gas. A solution of DBU in anhydrous DCM (0.13 g, 0.84 mmol) was added to the stirring solution under nitrogen gas atmosphere. The excess amount of acetic acid in DCM was added to the reaction mixture to quench the reaction. The P407-PBP was purified by precipitation into ether and dried under vacuum to obtain a white powder product. P407-PBP hydrogel formulations were made by dissolving powdered polymers in deionized water and adding CPEs similarly as the P188 formulation.

**Rheological Measurements**. The gelation temperature and mechanical properties [i.e., the storage (G′) and loss (G”) moduli] of a hydrogel formulation were quantified by linear oscillatory shear rheology measurements using TA instrument Discovery HR-2 (New Castle, DE). The rheology experiments were run under temperature sweep mode at 5% strain, and the frequency of 10 rad/s. Gelation temperature was taken as the temperature at which the storage modulus (G′) becomes greater than the loss modulus (G″). To confirm that the rheology measurement conditions were within the linear viscoelastic region of the material, the gelation temperature obtained from rheological experiments were compared with a published water bath method. The results obtained from two methods were consistent (Table S1) [39]. The shear viscosity was studied by measuring flow curves recorded at shear rates from 0 to 100 s^−1^ at room temperature.

**Small-angle neutron scattering measurement (SANS)**. To investigate the P188 micellar form and structure factors with various CPE agents, SANS experiments were performed at the Spallation Neutron Source of Oak Ridge National laboratory with the EQ-SANS instrument, which is a time-of-flight SANS instrument [40]. Two configurations of the instrument were employed at the sample-detector distances of 4 m and 2.5 m with wavelength bands with λ _min_ = 10 Å and 2.5 Å, respectively, covering momentum transfer range 0.006 Å^−1^ < *q* < 0.4 Å^−1^. Here, *q* is the momentum transfer value defined as q=4πsinθ/λ,where2θ is the scattering angle and λ is the wavelength of neutrons (q=4πsinθ/λ,2θ is the scattering angle). All samples were measured using quartz cells with 1 mm path length. (Hellma; Germany). An empty cell was also measured as a background. Data reduction was performed using the MANTID software package which corrects for detector pixel sensitivity and background subtraction [41]. The reduced data were scaled into absolute units by using a porous silica standard as a reference. [42] All SANS measurement were performed at temperatures between 20 °C and 60 °C, in increments of 5 °C. Analysis of the reduced SANS data was done with build-in functions in SAS View 5.0.5 software.

**DLS Measurements.** The hydrodynamic diameter of micelles in the presence and absence of different SDS were measured using a Malvern Zetasizer Nanoseries Nano-ZS instrument equipped with He–Ne laser (λ = 632.8 nm) at 60 °C.

**Ex vivo TM permeation.** The Ex vivo TM permeation rate of antibiotics was determined with auditory bullae harvested from healthy chinchillas. All formulations (200 μL) were applied into the bullae kept at 37 °C and deposited onto the TMs. The bullae were placed with the ear canal side facing up in a 12-well plate with 3 mL phosphate buffered saline (PBS) in each well. Permeation of ciprofloxacin across TM into the receiving chamber after 0.5, 1, 2, 6, 12, 24 and 48 h at 37 °C was quantified using liquid chromatography mass spectrometry (LC-MS). Detailed information regarding TM harvesting, electrical resistance measurement and configuration of the ex vivo permeation experiment can be found in our previous study [43].

***NTHi* OM chinchilla model and pharmacokinetics.** All procedures and manipulations were performed using sedation analgesia under isoflurane chamber induction followed by mask inhalation of 1–3% isoflurane in accordance with approved IACUC protocols at Boston Children's Hospital. Baseline plasma samples were obtained through the cephalic sinus via an insulin syringe (29-gauge, 0.5-inch) 24 h before bacterial inoculation under anesthesia. Isolates of *NTHi* grown to the mid-log phase were diluted in Hanks' balanced salt solution (HBSS), and about 25–75 CFU in 100 ml were directly introduced into each middle ear through the dorsal aspect of the tympanic bullae under aseptic conditions. Once the middle ear infection was confirmed using methods described previously, the chinchillas were administrated with 200 μL test formulations via a soft catheter (20-gauge, 1.8-inch) on to the TM through their external ear canal [16].Then the middle ear fluids (MEF) were obtained with a 22-gauge angiocatheter connected syringe after 2, 6, 24, 48, and 168 h based on the previously described methods [16].The serial 10-fold dilutions of MEF were prepared in HBSS and then one hundred microliters of each dilution were plated onto the blood agar for bacterial counting. Serial blood samples were also obtained through the cephalic sinus during the experiment to determine systemic drug levels. Plasma samples were obtained after centrifugation at 14,000 × rpm for 30 min at 4 °C and then filtered (0.22 μm filter) before further analysis by HPLC.

The commonly used thermoresponsive material P407 was not used as a comparison here since the gelation properties of P407 can be impaired by the presence of CPE agents, resulting in inadequate antibiotic flux across the TM [16,43]. In this study, OM was defined as more than zero colony-forming units (CFU) in middle ear fluid (MEF), and a reduction of the bacterial count by 99.9% (i.e., a 3-log reduction) was considered an indication of cure.

**Histopathology.** Hydrogel formulations were administered onto the tympanic membrane of live healthy/OM chinchillas. After 7 days later, they were euthanized as described elsewhere. The TMs were then excised and immediately fixed in 10% neutral buffered formalin overnight. The sectioned TMs (∼10 μm thick) were stained with hematoxylin and eosin and evaluated by light microscopy in a blinded fashion.

**Statistical analysis.** The results are reported as averages and standard deviations, and the differences among treatments were calculated based on an analysis of variance (ANOVA) and a post-hoc Duncan test with a confidence level of 95%. These analyses were carried out using statistical analysis software (SPSS, IBM Corporation, Armonk, NY, USA).

**Study approval.** All experimentation was conducted in accordance with protocols (20-12-4301R) approved by Boston Children's Hospital Institutional Animal Care and Use Committee and are in accordance with the National Institutes of Health Office of Laboratory Animal Welfare policies and laws.

## Author contribution statement

Zipei Zhang- Performed the experiments; Analyzed and interpreted the data; Wrote the paper.

Xiyu Li-Performed the experiments.

Changwoo Do -Performed the experiments; Analyzed and interpreted the data; Contributed reagents, materials, analysis tools or data; Wrote the paper.

Daniel S. Kohane - Conceived and designed the experiments; Analyzed and interpreted the data; Wrote the paper.

## Data availability statement

Data will be made available on request.

## Declaration of competing interest

The authors declare that they have no known competing financial interests or personal relationships that could have appeared to influence the work reported in this paper.
