# Supplementary material for: Enhancement of polymer thermoresponsiveness and drug delivery across biological barriers by addition of small molecules
Source: Heliyon. 2023 Jun 7;9(6):e16923. doi: 10.1016/j.heliyon.2023.e16923 (PMC10360936; doi:10.1016/j.heliyon.2023.e16923)
Supplement: Multimedia component 1 [file mmc1.docx]

**SUPPLEMENTARY MATERIAL**

**Scattering Light Density** (**SLD) profile modelling for the micelles**

The scattering intensity from pure P188 solution and P188-LIM solution observed at 60 °C (Figure 3C) was fitted using the micelle scattering model with the PPO blocks forming the micellar core and PEO blocks constituting the corona. The scattering intensity for monodisperse micelles can be written as,

$$I\left( q \right)=nP_{SLD}\left( q \right)S\left( q \right)+B$$

Where *n* is the number density of the micelle and *B* is constant term that represents the incoherent background. The form factor F(q) describes the shape and size of particles, and the structure factor S(q) describes the interactions between particles. In our analysis on the 2 wt % polymer solution, we assumed a dilute condition where S(q) may be approximated as 1. Here, we approximated the block copolymer micelle as a sphere with smoothly decaying SLD, which may embrace several key features of micelles: 1) spherical symmetry, 2) penetration of solvent molecules, and 3) corona-like polymer chains.

We assumed the SLD profile to have exponential decaying function as:

$$\rho\left( r \right)=\left( \rho_{core}-\rho_{solv} \right)\times e^{-\nu\cdot r/t}+\rho_{solv}$$

, where $\rho_{core}$ is the SLD of the micelle core which is assumed to be the SLD of the block copolymer itself, $\rho_{solv}$ is the SLD of the solvent (D_2_O), $\nu$ and t are the parameters that define the shape of the decaying function. The form factor of a particle with symmetric SLD profile ($\rho(r)$) can be calculated by:

$$P_{SLD}\left( q \right)=\frac{1}{V_{particle}}\left| 4\pi\int_{0}^{\infty} \rho\left( r \right)\frac{\sin\left( qr \right)}{qr^{2}}r^{2}dr \right|^{2}$$

**Table S1.** Gelation temperatures of P188 formulations obtained with a rheometer and by a water bath method (3CPE: 2% LIM, 1% SDS and 0.5% BUP).[1]

| **Formulations** | **rheology measurement** | **water bath method** |
| --- | --- | --- |
| 45% P188 | 62.0 °C | 63.5 °C |
| 45% P188-3CPE | 25.0 °C | 26.0 °C |

**Figure S1.** Rheology of different poloxamers as a function of temperature. None of the tested poloxamers except P188 formed gels in the temperature range 10° to 75°C.

**Figure S2.** Rheology of different poloxamers with 3CPE, as a function of temperature. None of the tested poloxamers-CPE combinations except P188 formed gels in the temperature range 10° to 75°C. Data for P188 with 3CPE are in Figure 1.

**Figure S3.** Effect of (A) SDS and (B) BUP concentration on mechanical properties of 45% P188, as a function of temperature.

**Figure S4.** Particle size of 5% P188 at various SDS concentrations and temperature at 60 ℃.

**Figure S5**. Tympanic membranes excised from healthy chinchillas 21 days.

**Supplementary References**

[1] M.-O. Yun, H.-G. Choi, J.-H. Jung, C.-K. Kim, Development of a thermo-reversible insulin liquid suppository with bioavailability enhancement, International journal of pharmaceutics 189(2) (1999) 137-145.
